# Atypical hepatitis B surface antigen escape mutations in an HIV-positive patient in South Africa

**DOI:** 10.4102/sajid.v41i1.818

**Published:** 2026-06-30

**Authors:** Menzi B. Nkosi, Marije van Schalkwyk, Tongai G. Maponga, Yoliswa Z. Chili, Devon Muir, Paula R. Delport, Susan S. Hugo, Zahiera Ismail, Jantjie Taljaard, Gert U. van Zyl, Wolfgang Preiser

**Affiliations:** 1 Division of Medical Virology, Department of Pathology, Faculty of Medicine and Health Sciences, Stellenbosch University, Cape Town, South Africa; 2 Tygerberg Business Unit, National Health Laboratory Service, Tygerberg Hospital, Cape Town, South Africa; 3 Division of Infectious Diseases, Department of Medicine, Faculty of Medicine and Health Sciences, Stellenbosch University, Cape Town, South Africa

**Keywords:** hepatitis B virus, HIV, antiretroviral therapy, immune escape mutations, viral reactivation, whole-genome sequencing

## Abstract

A 44-year-old woman with human immunodeficiency virus (HIV), chronic kidney disease and chronic diarrhoea presented with worsening gastrointestinal symptoms and renal impairment. Despite reported adherence, she had HIV viraemia and atypical hepatitis B serology (absence of hepatitis B core antibody and co-existence of hepatitis B surface antigen (HBsAg) and hepatitis B surface antibody) with high hepatitis B virus DNA. Whole genome sequencing of hepatitis B virus was performed using Oxford Nanopore Technologies sequencing on the Oxford Nanopore Technologies GridION (ONT, Oxford, United Kingdom). Genotype A infection with HBsAg immune escape mutations D144G/G145E was identified, without drug-resistance mutations.

**Contribution:**

Findings are most consistent with hepatitis B virus reactivation following prior natural infection and subsequent loss of immune control.

## Case presentation

**FIGURE 1:**
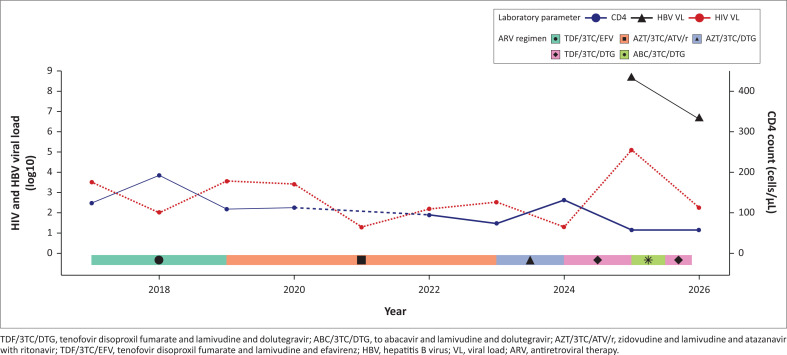
Longitudinal trends in HIV viral load and CD4 count with corresponding antiretroviral therapy regimens, 2018–2025.

A 44-year-old woman living with human immunodeficiency virus (WLHIV)^1^ with a known history of chronic kidney disease since 2017 and chronic diarrhoea since October 2023 presented in late February 2025 with worsening gastrointestinal symptoms and an acute-on-chronic renal impairment. The patient was managed as a case of *Clostridium difficile* infection and commenced on oral Vancomycin. She had initiated antiretroviral therapy (ART) in 2012 on tenofovir disoproxil fumarate and lamivudine and efavirenz (TDF/3TC/EFV) and was switched to second-line ART with zidovudine and lamivudine and atazanavir and ritonavir (AZT/3TC/ATV/r) in 2019 because of virological failure. She tested negative for hepatitis B surface antigen (HBsAg) at the time, and her HIV viral load was suppressed soon thereafter. Atazanavir was replaced with dolutegravir in 2022, and in 2023, she was diagnosed with pulmonary tuberculosis, which was treated with a standard 6-month course of Mycobacterium tuberculosis (*M. tb*) therapy, after which she achieved clinical resolution. She was subsequently switched to tenofovir disoproxil fumarate and lamivudine and dolutegravir (TDF/3TC/DTG) and on 26/03/2024 to abacavir and lamivudine and dolutegravir (ABC/3TC/DTG) regimen to protect against further renal injury (Table 1). One year later, despite self-reported adherence, the patient exhibited an HIV viral load of log_10_ 4.33 and a cluster of differentiation 4 (CD4) count of 131 cells/mm^3^ (Figure 1; Table 2); however, intermittent gaps in hepatitis B viral load (HBVL) and CD4 measurements limited the completeness of the longitudinal analysis.

**TABLE 1:** Timeline of antiretroviral therapy regimen changes and clinical events highlighting treatment adjustments in response to virological failure, renal function and tuberculosis co-infection.

Year	Event	Treatment
2012	Initiation of ART	TDF/3TC/EFV
2019	Switched to second-line ART because of virological failure	AZT/3TC/ATV/r
2022	Regimen modification	ATV replaced with DTG
2023	Diagnosed with pulmonarytuberculosis	Standard *M. tb*. treatment continued for 6 months
Post-2023	ART adjusted	Switched to TDF/3TC/DTG
2024	ART switched again to protect renal function	Switched to ABC/3TC/DTG
2025	ART switched once renal function improved	Switched to TDF/3TC/DTG

ART, antiretroviral therapy; TDF/3TC/DTG, tenofovir disoproxil fumarate and lamivudine and dolutegravir; ABC/3TC/DTG, to abacavir and lamivudine and dolutegravir; AZT/3TC/ATV/r, zidovudine and lamivudine and atazanavir with ritonavir; TDF/3TC/EFV, tenofovir disoproxil fumarate and lamivudine and efavirenz.

**TABLE 2:** Longitudinal HIV and hepatitis B virus virological markers, CD4 T-cell counts and antiretroviral therapy.

Year	HIV_VL_copies/mL	log10	HBVL (IU/mL)	log10	CD4 (cells/μL)	Treatment
2018	100	2.00	n/a	n/a	192	TDF/3TC/EFV
2019	3614	3.56	n/a	n/a	109	AZT/3TC/ATV/r
2020	2474	3.39	n/a	n/a	113	AZT/3TC/ATV/r
2021	19.9	1.30	n/a	n/a	n/a	AZT/3TC/ATV/r
2022	146	2.16	n/a	n/a	94	AZT/3TC/ATV/r
2023	331	2.52	n/a	n/a	73	AZT/3TC/DTG
2024	19.9	1.30	n/a	n/a	131	TDF/3TC/DTG
2025	123 595	5.09	418 740 418	8.62	57	ABC/3TC/DTG
2025	173	2.24	4 142 578	6.62	57	TDF/3TC/DTG

n/a, not applicable; HBV, hepatitis B virus; HBVL, hepatitis B viral load; TDF/3TC/DTG, tenofovir disoproxil fumarate and lamivudine and dolutegravir; ABC/3TC/DTG, to abacavir and lamivudine and dolutegravir; AZT/3TC/ATV/r, zidovudine and lamivudine and atazanavir with ritonavir; TDF/3TC/EFV, tenofovir disoproxil fumarate and lamivudine and efavirenz.

Routine inpatient workup included hepatitis B virus (HBV) markers and revealed positive results for HBsAg and hepatitis B surface antibody (anti-HBs) at 37 IU/mL with a negative hepatitis B core IgM (IgM anti-HBc) and core total antibody (anti-HBc). Extended testing found a positive hepatitis B e antigen (HBeAg) and negative hepatitis B e antibody (anti-HBe). The HBVL was log_10_ 8.4 IU/mL. An elevated alkaline phosphatase was observed at 116 U/L (normal range of 42 U/L – 98 U/L); however, the rest of her liver function tests was within normal limits. She had no history of prior HBV vaccination and was asymptomatic for a viral hepatitis syndrome with normal aminotransferase levels and no fibrosis detected on FibroScan^®^.

## Research methods and design

All routine laboratory tests were conducted by the National Health Laboratory Service at Tygerberg Hospital at the Divisions of Haematological Pathology, Chemical Pathology and Medical Virology. Serological tests were done on the Roche cobas^®^ e 601 instrument (Roche Diagnostics, Mannheim, Germany). Quantitative PCR testing was done on the Abbott Alinity™ m Diagnostics System (Abbott Molecular Inc., Des Plaines, IL, United States).

Whole-genome sequencing of HBV was performed on a plasma sample obtained on 07 March 2025 using Oxford Nanopore Technologies (ONT) sequencing on the ONT GridION (ONT, Oxford, United Kingdom) using a protocol established by Tshiabuila et al. in 2024.^2^ Analysis revealed that the patient was infected with HBV genotype A. Consensus sequences to identify HBV genotype A were generated using Genome Detective, an automated system for virus identification from high-throughput sequencing data analysed for the presence of known drug-resistance mutations^3^ and the Stanford University HBVseq database.^4^ Drug-resistance mutations and vaccine escape mutations were assessed using the online tool Geno2pheno (hbv) 2.0 (Max Planck Institute for Informatics, Saarbrücken, Germany). No known drug resistance associated mutations were detected. Results were further confirmed by alignment of the overlapping polymerase and HBsAg regions with other genotype A sequences from GenBank using Geneious Prime software (Biomatters Ltd, Auckland, New Zealand) (Accession number PX931884).^2^

Mutations resulting in D144G and G145E substitutions were detected in the ‘a’ determinant of the surface gene (S gene). These are rare mutations previously described in only a single recipient of an allogeneic haematopoietic stem cell transplant from an HBV-vaccinated donor^5^ as well as in two mother-infant pairs, whereby infants were infected vertically with HBV despite having received HBV immunoprophylaxis.^6^

## Discussion

In South Africa, the estimated prevalence of HBsAg seropositivity among people living with HIV (PWH) is estimated to be between 5% and 17%.^7^ HIV and HBV co-infection increases the risk of mortality as a result of accelerated progression of liver disease – including fibrosis, cirrhosis and hepatocellular carcinoma.^7^ Patients co-infected with HIV are more likely to access medical care, which may include inadvertent co-treatment of HBV if antiretroviral compounds with HBV activity are used.^8^ This co-infection is also associated with three notable phenomena:

- Occult HBV infection – defined as the presence of HBV DNA in the liver, in the absence of detectable HBsAg, with or without HBV DNA detection in the blood.^9^
- High HBV DNA loads – HIV-related depletion of CD4+ T lymphocytes, together with CD8+ T-cell dysregulation, promotes increased transcription of covalently closed circular DNA, thereby facilitating higher HBV replication.^9^
- Seroreversion to HBsAg positivity – HIV-induced immunosuppression impairs HBV-specific T- and B-lymphocyte responses, resulting in loss of immune surveillance and re-emergence of HBsAg.^9^

Given the above clinical outcomes and the shared transmission routes, routine HBV screening in PWH is essential. The 2023 South African antiretroviral clinical guidelines recommend testing for HBsAg at HIV diagnosis, before modifying ART regimens, prior to any changes in HBV-active antivirals, during pregnancy, and in cases of unexplained liver function abnormalities.^10,11^ With regard to the patient described here, explanations for an initial absence in 2019 but subsequent detection of HBsAg include: (1) HBV infection acquired after 2019, (2) a false-negative HBsAg result in 2019 or (3) reactivation of previously resolved HBV infection because of immunosuppression.

In HIV and HBV co-infection, antiretroviral regimens with dual activity, most notably tenofovir disoproxil fumarate, tenofovir alafenamide, lamivudine and emtricitabine, are recommended. The most recent South African antiretroviral clinical guidelines advocate for tenofovir-based therapy as first-line therapy because of its dual efficacy, while entecavir is advised in cases of tenofovir-associated toxicity. Lamivudine monotherapy is discouraged because of the increased risk of acquiring drug-resistance mutations.^10,11,12^ In this individual, immune escape mutations arose in the context of significant HIV-related immunosuppression, with the most recent HIV viral load suggesting inadequate adherence. Chronic diarrhoea is unlikely to significantly impair drug absorption or reduce antiretroviral exposure; such effects have predominantly been described in the context of short bowel syndrome.^13,14^ The 6-month course of empiric *M. tb*. treatment also unlikely contributed to decreased antiretroviral exposure in this case, as the patient was prescribed twice daily dolutegravir.^15^ HBsAg escape variants have previously been reported under two conditions: Reactivation after natural infection and ensuing immune control, associated with a positive anti-HBs status, or vaccination during the incubation period of primary HBV infection.^16^ Since this patient reports never having been vaccinated against HBV, viral reactivation during immune suppression – associated with poor adherence to an ART regimen that would also be active against HBV infection – is the most likely explanation. Reactivation is less likely explained by HBV drug resistance, as no resistance-associated mutations were identified. Notably, following the initiation of alternating antiretroviral regimens – abacavir and lamivudine and dolutegravir and tenofovir disoproxil fumarate and lamivudine and dolutegravir – tailored to the degree of renal impairment (while awaiting access to tenofovir alafenamide), a concurrent decline in both human immunodeficiency virus and hepatitis B virus viral loads was observed. Despite the clinical context of long-term lamivudine monotherapy, mutations such as the M204V/I substitution in the Tyrosine-Methionine-Aspartate-Aspartate (YMDD) catalytic domain of the reverse transcriptase enzyme – classically associated with lamivudine exposure and typically preceding HBV breakthrough – were absent. This, however, may have been because of decreased drug pressure and archiving of mutations, as seen in cases of poor adherence, which is of critical importance in this case, whereby adherence to ART was self-reported, which is often unreliable.^17^

Immune escape mutations in HBV most often involve substitutions in the S gene, particularly within the ‘a’ determinant region, which is the primary target of neutralising anti-HBs antibodies. A common example is the glycine substitution at amino acid 145. The D144G and G145E substitutions identified in this case similarly alter antigenic epitopes, preventing recognition by neutralising antibodies. These mutations may arise naturally during chronic HBV infection, under selective immune pressure from active or passive immunisation, or in association with antiviral therapy as a result of the overlap between the S and polymerase genes. Clinically, immune escape mutations can cause diagnostic challenges, depending on the assay used for HBsAg detection, and may present as breakthrough infection in vaccinated individuals or reactivation in those with prior natural immunity.^18^

The atypical serological profile seen in this case of HBsAg positivity without detectable anti-HBc has been reported elsewhere. Brousseau et al. reported that long-term persistence of HBsAg with delayed seroconversion to anti-HBc can occur in immunosuppressed individuals.^19^ Consistent with this observation, population-based studies have documented HBsAg positivity in the absence of anti-HBc in up to 13% of PWH in Botswana and approximately 10% of PWH in Brazil.^20^ In the present case, the absence of anti-HBc may plausibly be attributed to HIV-related immunodeficiency, given the patient’s uncontrolled HIV viraemia, low CD4+ T lymphocyte count of 131 cells/mm^3^ and chronic diarrhoea. An important clinical dilemma presented by this case is whether this constitutes a new HBV infection or reactivation of chronic HBV infection. Yotsuyanagi et al.^21^ reported that HBsAg clearance can take up to 12 months following acute HBV infection, complicating such distinctions.

Possible explanations for the absence of hepatitis B core antibody and co-existence of hepatitis B surface antigen and hepatitis B surface antibody include: (1) Reactivation of HBV infection during severe immune suppression, a phenomenon well-documented in individuals who had previously cleared the virus and developed anti-HBs^16^; (2) undisclosed vaccination concurrent with HBV infection, typically occurring when vaccination is administered during the incubation period and (3) transmission of a pre-existing immune escape strain. Among these, reactivation of latent HBV infection is the most plausible explanation in this case, particularly within the context of HIV-associated immunosuppression; evidenced by high HBV DNA levels, the presence of atypical surface antigen escape mutations (D144G and G145E) and anti-HBs seropositivity without detectable anti-HBc. Given that the patient was born prior to the implementation of universal childhood HBV immunisation and in the absence of a vaccination history, undisclosed vaccination or acute infection with a rare immune escape variant are less likely. This case underscores some of the diagnostic and therapeutic challenges posed by HIV and HBV co-infected individuals and illustrates the need for proper management of both in clinical and laboratory settings.
